# 5-Hydroxymethylcytosine is an essential intermediate of active DNA demethylation processes in primary human monocytes

**DOI:** 10.1186/gb-2013-14-5-r46

**Published:** 2013-05-26

**Authors:** Maja Klug, Sandra Schmidhofer, Claudia Gebhard, Reinhard Andreesen, Michael Rehli

**Affiliations:** 1 Department of Internal Medicine III, University Hospital Regensburg, D-93042 Regensburg, Germany; 2 Regensburg Centre for Interventional Immunology (RCI), D-93042 Regensburg, Germany; 3 German Cancer Research Center (DKFZ), D-69120 Heidelberg, Germany

**Keywords:** Epigenetics, active DNA demethylation, differentiation

## Abstract

**Background:**

Cytosine methylation is a frequent epigenetic modification restricting the activity of gene regulatory elements. Whereas DNA methylation patterns are generally inherited during replication, both embryonic and somatic differentiation processes require the removal of cytosine methylation at specific gene loci to activate lineage-restricted elements. However, the exact mechanisms facilitating the erasure of DNA methylation remain unclear in many cases.

**Results:**

We previously established human post-proliferative monocytes as a model to study active DNA demethylation. We now show, for several previously identified genomic sites, that the loss of DNA methylation during the differentiation of primary, post-proliferative human monocytes into dendritic cells is preceded by the local appearance of 5-hydroxymethylcytosine. Monocytes were found to express the methylcytosine dioxygenase Ten-Eleven Translocation (TET) 2, which is frequently mutated in myeloid malignancies. The siRNA-mediated knockdown of this enzyme in primary monocytes prevented active DNA demethylation, suggesting that TET2 is essential for the proper execution of this process in human monocytes.

**Conclusions:**

The work described here provides definite evidence that TET2-mediated conversion of 5-methylcytosine to 5-hydroxymethylcytosine initiates targeted, active DNA demethylation in a mature postmitotic myeloid cell type.

## Background

DNA methylation is a frequent epigenetic modification that restricts the activity of regulatory elements, including cell type-specific gene promoters and enhancers. In mammals, methylated cytosines (5mC) mainly occur in the context of CpG dinucleotides and the targeted setting and erasure of the methylation mark is crucial for the silencing of repetitive and potentially harmful elements and for the proper execution of essential regulatory programs including embryonic development, X-chromosome inactivation, parental imprinting as well as cellular differentiation [1,2]. While the process of cytosine methylation, which is catalyzed by a group of DNA methyl-transferases (DNMTs) is well characterized, the exact mechanisms facilitating the erasure of DNA methylation in mammals remain less clear and the proposed existence of active enzymatic demethylation processes has been a matter of controversy over the last decades [3].

Recent pioneering work has identified the family of Ten-Eleven-Translocation proteins (TET1-3) that catalyze the conversion of 5mC to 5-hydroxy-methylcytosine (5hmC) in mammalian cells [4], and has prompted speculations that these enzymes are involved in DNA demethylation processes [5,6]. On the one hand, 5hmC could interfere with maintenance methylation and induce a passive demethylation process. On the other hand, TET enzymes may also initiate active demethylation processes through repair-associated mechanisms [7].

Global DNA demethylation is observed during early embryonal development in particular in zygotes and primordial germ cells and 5hmC has been detected in both pathways [8,9]. The initial massive erasure of 5mC in primordial germ cells, however, appears to be a TET-independent, passive process that is likely controlled by the downregulation of UHRF1, which facilitates the recruitment of the maintenance DNA-methyltransferase DNMT1 to nascent hemimethylated DNA at the replication fork [10]. In the zygote, however, TET3 mediated conversion of 5mC to 5hmC is essential for the reprogramming of the zygotic paternal DNA after fertilization [11-13]. 5hmC is then gradually replaced by unmethylated cytosines during preimplantation development, suggesting that the erasure of 5hmC in zygotes is also a DNA replication-dependent passive process [12].

Another member of this family (TET2) directly affects myelopoiesis and diverse myeloid malignancies (including myelodysplastic syndromes, chronic myelomonocytic leukemia, myeloproliferative neoplasms, and acute myeloid leukemia) are frequently associated with mutations in this gene [14-16]. Targeted disruption or knockdown of *TET2 *results in reduced levels of 5hmC and affects self-renewal and differentiation of hematopoietic stem cells [17-20]. However, the exact mechanisms that contribute to disease pathology are currently unknown.

Here we provide direct evidence that the TET2-dependent conversion of 5mC to 5hmC is required for active DNA demethylation in primary human monocytes. Similar processes are likely to occur in other myeloid (progenitor) cells and the reduced ability to erase DNA methylation at critical regulatory sites in cases with TET2 loss-of-function mutations may therefore contribute to disease pathology.

## Results and discussion

**Figure 1:**
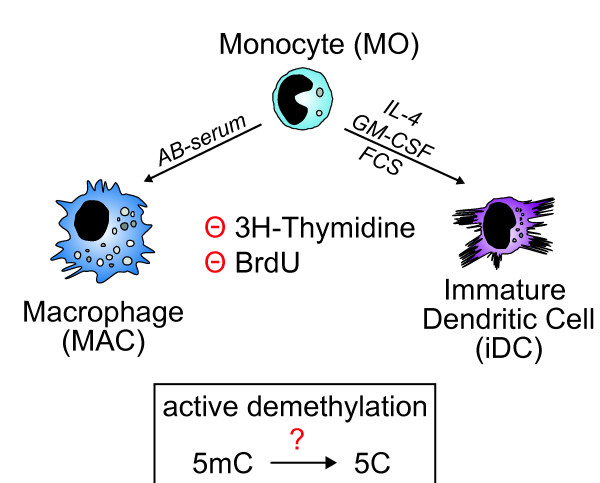
**Monocyte differentiation *in vitro***. Schematic presentation of the postmitotic differentiation model of *in vitro *monocyte differentiation. Monocytes do not proliferate (as demonstrated by the lack of nucleotide incorporation) and DNA demethylation therefore requires an active process.

Many previous studies focused on proliferating cell types that were mitotically arrested to distinguish between active and passive DNA demethylation mechanisms. Using methyl-CpG-immunoprecipitation (MCIp) and a global microarray-based approach to detect differences in DNA methylation [21,22], we recently identified a number of actively demethylated regions in a natural setting of post-mitotic cells: the differentiation of human peripheral blood monocytes into monocyte-derived macrophages or dendritic cells (Figure 1). DNA demethylation events were highly reproducible and paralleled or followed the appearance of 'activating' histone modifications, suggesting that a proposed DNA demethylation machinery is recruited as part of other chromatin-modifying processes associated with gene activation or transcriptional priming [23]. However, the exact mechanisms leading to DNA demethylation in human monocytes remain unclear.

**Figure 2:**
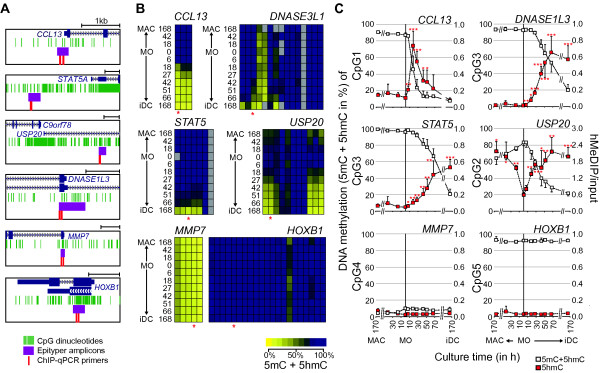
**5hmC deposition precedes active DNA demethylation in human monocytes**. (**A**) Positions of regions (purple) measured by MALDI-TOF analysis of bisulfite converted DNA (MassARRAY, in **B**) and of primers (red) used for hMeDIP qPCR (in **C**) are shown relative to positions of CpG dinucleotides (green) and neighboring genes (blue). Tracks were generated using the UCSC Genome Browser. (B) MassARRAY analysis of bisulfite-converted DNA at four loci that show active DNA demethylation during monocyte to DC differentiation, as well as for two control regions (values are mean of *n*≥4). Data are presented as heatmaps. The methylation content (including both 5mC and 5hmC) is indicated by coloring (yellow: no methylation, dark blue: 100% methylation) with each box representing a single CpG dinucleotide and each row representing the succession of CpGs measured. Grey boxes indicate CpGs that were not detected by MALDI-TOF MS. Red asterisks mark the CpGs that are shown in (C). Methylation ratios of single CpG units for individual donors are also provided in Table S2 in Additional File 2. (C) Dynamics of DNA methylation (5mC + 5hmC) and hydroxymethylation (5hmC) during monocytic differentiation. DNA methylation levels of single CpGs as measured by MassARRAY (open squares) are compared with 5hmC enrichment (measured by hMeDIP, red squares) at the same loci shown in (B) (*n*≥4, values are mean + or - SD). Exact genomic positions of analyzed CpG residues are given in Table S3 in Additional File 3.

Recent studies have implicated the family of Ten-Eleven-Translocation proteins (TET1-3) in active demethylation processes via the generation of 5hmC. To test whether TET proteins might be involved, we studied the appearance of 5hmC at previously defined sites of active DNA demethylation [23] in time-courses of differentiating human monocytes and compared the appearance of 5hmC (measured using hMeDIP) with traditional measurements of bisulfite treated DNA (which globally converts cytosines to uracil except 5mC and 5hmC) using mass spectrometry as well as 5mC levels measured by MeDIP. Figure 2 summarizes the results for six different genomic regions. These loci (schematically illustrated in Figure 2A) included four regions showing DNA demethylation with either fast (CCL13, USP20) or slow kinetics as well as two control regions characterized by constitutive DNA methylation (HOXB1) or demethylation (MMP7) during the time courses. As shown in Figure 2C, the local erasure of DNA methylation (5mC and 5hmC as detected by mass spectrometry) was always found to correlate with the synchronous appearance of 5hmC (as measured by hMeDIP). At the *CCL13 *promoter both 5mC and 5hmC disappeared at later stages of DC differentiation, suggesting that the erasure of methylation marks might proceed to completeness (as observed at CpG dinucleotide 1) in this case. Since measurements of bisulfite treated DNA do not distinguish between 5mC and 5hmC, we also followed local DNA methylation levels using MeDIP (Figure S1 in Additional File 5). While the 5mC antibody appeared less sensitive compared to the 5hmC antibody, we also observed demethylation of the four loci using this method. The local conversion of 5mC to 5hmC was further confirmed by an independent approach utilizing 5hmC-specific glycosyltransferase for 5hmC detection by glycosylation-sensitive restriction (Figure S2 in Additional File 5). These results clearly established that 5hmC appears at actively demethylated sites during monocyte differentiation.

**Figure 3:**
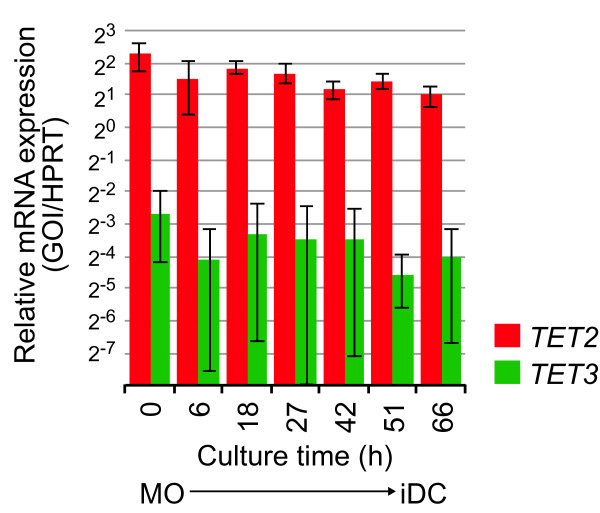
**TET2 is expressed in human monocytes**. The expression profile of *TET2 *during monocyte differentiation into dendritic cells is shown. Quantitative RT-PCR results are shown relative to *HPRT1 *expression and represent mean values ± SD (*n*=6). *TET3 *levels were considerably lower and no mRNA expression was detected for *TET1 *in monocytes or monocyte-derived cells.

To study a causal relationship between TET2 enzymes and DNA demethylation, we analyzed mRNA expression of TET genes during monocyte differentiation and found that monocytes and monocyte-derived cells primarily expressed *TET2 *(Figure 3). Expression of *TET1 *was undetectable and expression of *TET3 *was much weaker and less reproducible (Figure 3). In contrast to TET2 (see below) we were also unable to detect TET1 or TET3 by western blotting using commercial antibodies. These data established *TET2 *as the candidate enzyme for the local oxidation of 5mC in monocytes.

**Figure 4:**
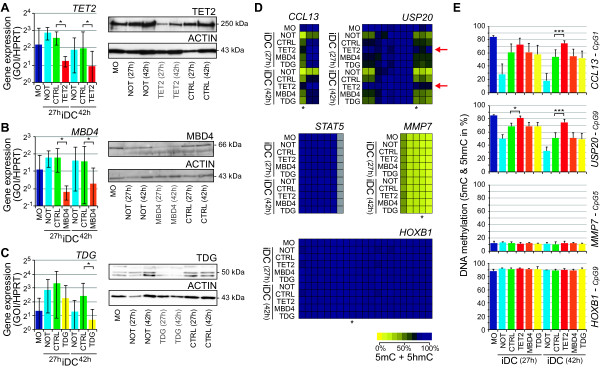
**TET2 is required for active DNA demethylation in human monocytes**. (**A-C**) mRNA (left panels) and protein expression (right panels) of TET2, MBD4, or TDG in monocytes left untreated or transfected with the corresponding TET2-, MBD4-, TDG-siRNA, or control siRNA after 27 h and 42 h of differentiation culture. qRT-PCR results were normalized to HPRT1 expression (*n*≥4, values are mean ± SD, * *P *<0.05 Student's T-test, paired, two-sided). Protein levels of TET2, MBD4, or TDG were analysed using western blotting (results are representative of *n*=3 independent experiments). (**D**) MassARRAY analysis of bisulfite-converted DNA at five loci that show active DNA demethylation during monocyte to DC differentiation, as well as for two control regions (values are mean of *n*≥4). Data are presented as heatmaps. The methylation content (including both 5mC and 5hmC) is indicated by coloring (yellow: no methylation, dark blue: 100% methylation) with each box representing a single CpG dinucleotide and each row representing the succession of CpGs measured. Grey boxes indicate CpGs that were not detected by MassARRAY. Asterisks mark the CpGs that are shown in (**E**). Red arrows mark TET2-siRNA treated samples that show a specific decrease in demethylation. (*DNase1L3 *methylation did not change during the first 42 h, but spectra were of low quality and are not shown.) Methylation ratios of single CpG units for individual donors are also provided in Table S4 in Additional File 4. (E) Bar charts for MassARRAY results of the indicated CpG residues of actively demethylated (*CCL13*, *USP20*) or control loci (*MMP7*, *HOXB1*). Values are mean ± SD (*n*≥4; * *P *<0.05, *** *P *<0.001 Student's T-test, paired, two-sided).

To test whether ablation of TET2 would affect the process of active DNA demethylation, we established a transient siRNA transfection protocol for freshly isolated human blood monocytes using control- or *TET2*-siRNA before culturing them under iDC culture conditions. Monocytes are generally difficult to transfect - as sensitive sentinels of the innate immune system, they respond to foreign nucleic acids including plasmid DNA and siRNAs, which affects differentiation and survival. We thus only studied 'early' time points (27 h and 42 h) after transfection where survival was largely unaffected by siRNA transfection (Figure S3A in Additional File 5). As shown by the reduced expression of the DC markers CD1a (FACS staining in Figure S3A and qRT-PCR in Figure S3B in Additional File 5) and *CCL13 *(qRT-PCR in Figure S3B in Additional File 5) the transfection did have an effect on the differentiation process. Concomitant, transient transfections also delayed DNA demethylation (see below), but since the process was clearly detected, the siRNA approach still allows addressing the demethylation mechanism. In addition to analyzing the effects of *TET2 *knockdown, we also established the siRNA-mediated knockdown of the two DNA glycosylases *MBD4 *and *TDG *that have previously been implicated in the removal of deaminated 5mC [7,24,25] or 5hmC via 5-Carboxylcytosine (5caC) [26,27] in other cellular systems. As shown in Figure 4A-C the average knockdown at 27 h or 42 h ranged between 25% and 60% and 5% and 40% of control siRNA transfected cells on RNA and on protein level, respectively.

To study the effect of siRNA knockdowns, DNA methylation levels were analyzed at 27 h or 42 h using mass spectrometry of bisulfite treated DNA. Results are shown as heatmaps for the entire regions (Figure 4D) and bar charts for selected individual CpGs (Figure 4E). Interestingly, the methylation pattern derived from bisulfite treated DNA after knockdown of *MBD4 *or *TDG *was undistinguishable from control siRNA treatment (Figure 4D and 4E). This indicates that neither of these two enzymes actively converts 5hmC, which is in line with previous observations [27]. Tet proteins were recently shown to metabolize 5hmC to 5-formylcytosine (5fC) and 5caC [28], which are both converted into uracil during bisulfite treatment [26,29]. Because we cannot distinguish 5fC/5caC from unmethylated cytosine residues after bisulfite treatment, we are currently not able to address whether *MBD4 *or *TDG *are active to 'complete' the demethylation process in primary monocytes that is initiated by TET2 mediated processing of 5mC into 5hmC and further into 5fC/5caC. Since human blood monocytes are (in contrast to monocyte-derived macrophages or dendritic cells) severely impaired in base and DNA double-strand break repair [30], the exchange of the two 5mC derivatives may be delayed. To test whether 5fC/5caC accumulate as the end product of the demethylation process at the *CCL13 *promoter, we analyzed the restriction efficiency of *Msp*I (which is inhibited by the presence of 5caC or 5fC [28]) at the site covering one of the demethylated CpGs. While MspI can also be inhibited by methylation of the outer C (5mCCGG) and thus may not allow the quantification of 5caC or 5fC, the fact that DNA from iDC (as well as from all knockdown experiments) could be efficiently cut with this restriction enzyme (Figure S4 in Additional File 5) suggests that the demethylation process is completed (5mC→5C) in iDC. *MBD4 *or *TDG *knockdown did not lead to a decrease in restriction efficiency, indicating that 5caC or 5fC do not accumulate at these sites in transfected monocytes. It is thus still unclear whether TDG, MBD4, or another enzyme initiates the last steps of the active demethylation process. Notably, a recent mass spectrometry study systematically identified readers in embryonic stem cells, neuronal progenitor cells, and brain for all known 5C derivates [31]. This study identified a number of additional DNA glycosylases (Neil1, Neil3), as well as helicases (Hells, Harp, Recql, and its homolog Bloom) binding specifically to hmC suggesting that this derivate may already attract DNA-repair enzymes and perhaps initiate DNA demethylation in differentiating monocytes.

The TET2-siRNA treatment, however, resulted in significantly different methylation patterns: the local loss of DNA methylation at the two loci showing rapid 5mC erasure (*CCL13 *and *USP20*) was significantly delayed in cells with reduced *TET2 *expression (Figure 4D and 4E), while control regions were unaffected and the late demethylation targets did not show any signs of methylation loss at these early time points. We also analyzed local 5hmC levels using hMeDIP (Figure S5A in Additional File 5), as well as glycosylation-sensitive restriction (Figure S5B in Additional File 5) and detected a significant reduction of 5hmC at demethylated regions only in *TET2*-siRNA-treated monocytes. These results clearly establish that differentiating monocytes require TET2 to initiate the active demethylation process.

As shown previously, DNA demethylation in primary monocytes is characterized by the parallel appearance of activating histone marks, such as mono- and dimethylation of H3K4 or acetylation of histones H3 and H4 [23], which are typical features of enhancers. This is also in line with the recent observation of dynamic deposition of 5hmC at differentiation-associated enhancers in other cellular systems [32]. The histone modifications likely follow the recruitment of DNA-binding factors that direct histone methyl- and/or acetyl-transferases to these sites [23]. The local appearance of 5hmC suggests that the modified histones or the same factors responsible for the modification of histones may also recruit the 5-methylcytosine dioxygenase TET2 to initiate DNA demethylation of newly activated and/or remodeled sites.

## Conclusions

Our data unequivocally show that the TET2-mediated conversion of 5mC to 5hmC is an essential intermediate in targeted, locus-specific active demethylation processes that are observed during the differentiation of non-dividing human monocytes. This function of TET2 may also be essential for the differentiation of earlier myeloid progenitor stages, as a significant proportion of myeloid dysplasia are characterized by loss-of-function mutations of TET2.

## Materials and methods

### Cells

Collection of blood cells from healthy donors was performed in compliance with the Helsinki Declaration. All donors signed an informed consent. The leukapheresis procedure, the subsequent purification of peripheral blood monocytes by density gradient centrifugation over Ficoll/Hypaque as well as the counter current centrifugal elutriation were approved by the local ethical committee (reference number 92-1782 and 09/066c). The generation of monocyte-derived dendritic cells and macrophages has been described previously [23].

### DNA isolation

Genomic DNA was prepared using the DNeasy Blood and Tissue Kit from Qiagen (Hilden, Germany).

### Mass spectrometry analysis of bisulfite-converted DNA

Sodium bisulfite conversion and quantitative analysis of DNA methylation using MALDI-TOF mass spectrometry (MassARRAY Compact MALDI-TOF, Sequenom, San Diego, CA, USA) was performed as described [23,33]. Primers for amplicon generation were described [23] or are listed in Table S1 in Additional File 1.

### hMeDIP & MeDIP

Enrichment of 5- methylcytosine (5mC) or 5-hydroxy-methylcytosine (5hmC) was analyzed by immunoprecipitation using 5-methylcytidine and 5-hydroxy-methylcytidine antibodies (Diagenode and Active Motif, respectively) essentially as described for 5mC in Mohn *et al. *[34]. Enriched DNA was purified with the PCR purification kit from Qiagen and quantified on a Realplex Mastercycler EP (Eppendorf, Hamburg, Germany) using the Quantifast SYBR Green PCR Kit (Qiagen) as indicated by the manufacturer. Primer sequences were previously described or are listed in Table S1 in Additional File 1.

### Glycosylation of 5hmC

Site-specific detection of 5hmC by glycosylation was done using the Quest 5-hmC detection kit (Zymo Research) following the manufacturer's instructions with modifications. After the glycosylation step (prolonged to 3 h), samples were cleaned using the DNA clean and concentrator kit (Zymo) and subsequently digested with 30 U *Msp*I (NEB), or 30 U *Hpa*II (NEB) at 37°C overnight. The fraction of glycosylated and therefore protected *Msp*I sites as well as the fraction of 5mC- and 5hmC-sensitive sites (determined using *Hpa*II restriction) at specific gene loci were quantified by qPCR using primers described in [23] or primers listed in Table S1 in Additional File 1.

### Quantitative RT-PCR

Total cellular RNA was isolated using the RNeasy Mini Kit (Qiagen) and reverse transcribed using Superscript II MMLV-RT (Promega, Mannheim, Germany). Real-time PCR was performed on a Realplex Mastercycler EP (Eppendorf, Hamburg, Germany) as described above. Primer sequences are listed in Table S1 in Additional File 1.

### Transfection of primary human monocytes

Peripheral blood monocytes were transfected using the Human Monocyte Nucleofector Kit from Lonza (Cologne, Germany). In brief, 6×10^6 ^cells were resuspended in 100 μL Nucleofector solution (Lonza) with 600 nM TET2-, MBD4-, TDG-, or control-siRNA (all from Thermo Scientific Dharmacon) and electroporated using the Nucleofector I device. Cells were cultured as described without the addition of antibiotics. Expression of targeted genes as well as DNA methylation was measured after 27 h or 42 h in culture.

### Western blotting

To follow knockdown efficiency on protein level, cells were harvested 27 h and 42 h after transfection, washed with PBS and lysed in 2x SDS-Lysis Buffer (20% Glycerin, 125mM Tris pH 6.8, 4% SDS, 10% 2-Mercaptoethanol, 0.02% Bromophenolblue). Lysates were boiled (95°C, 10 min) and 1.5 × 10^5 ^to 5 × 10^5 ^cells per lane separated on 8% or 10% polyacrylamide gels (Biometra Minigel Gelelectrophoresis device). Proteins were transferred to nitrocellulose membranes (Live Technologies, 0.45 μM pore size) using the Biometra Fastblot semi-dry blotter or the Biorad Mini Transblot Cell wet system according to the protein size. After 1 h of blocking in TBS-T with 5% dry milk at room temperature the membranes were incubated with either Anti-TET2 (1:2,000, a gift from O. Bernard), Anti-TDG (1:10,000, a gift from Primo Schär), Anti-MBD4 (1:2,000, from Diagenode) or Anti-actin (Sigma Aldrich) overnight at 4°C. Second antibody (Dako, Glostrup, Denmark) incubation was carried out at room temperature for 1 h. Flourescence signals were detected after exposure to ECL hyperfilm or using a fluorescence scanner (BioRad, Chemi Doc XRS+).

### Quantification of *Msp*I restriction efficiency

DNA from monocyte-derived dendritic cells (iDC, 100ng) was digested with *Msp*I plus *Hha*I or with *Hha*I alone (20 U each, New England BioLabs) overnight (approximately 16 h) at 37°C. The efficiency of *Msp*I cutting was measured by comparing the qPCR amplification of DNA fragments across the *Msp*I site in *Msp*I-digested and -undigested DNA samples.

## Abbreviations

5caC: 5-carboxylcytosine; 5fC: 5-formylcytosine; 5hmC: 5-hydroxymethylcytosine; 5mC: 5-methylcytosine; BrdU: 5-Bromo-2'-deoxy-uridine; iDC: immature dendritic cell; MO: monocyte; MAC: macrophage

## Competing interests

The authors declare that they have no competing interests.

## Authors' contributions

MK participated in study design, performed experiments, analyzed data, and aided in the manuscript preparation. SS performed experiments, analyzed data, and aided in the manuscript preparation. CG performed and analyzed experiments. RA participated in study design. MR conceived and coordinated the study, analyzed data, and drafted the manuscript. All authors read and approved the final manuscript.

## Supplementary Material

Additional file 1 **Table S1**. Word DocumentTitle of this dataset: Table S1, Oligonucleotide sequences used for PCRDescription of this dataset: Table S1 lists sequences of oligonucleotides for various PCR-based analyses. Click here for file

Additional File 2 **Table S2**. Excel FileTitle of this dataset: Table S2, EpiTYPER resultsDescription of this dataset: Table S2 lists MassARRAY EpiTYPER results. EpiTYPER methylation ratios of individual CpG units in 12 amplicons covering six distinct genomic locations are given for all knockdown samples of different donors. Click here for file

Additional file 3 **Table S3**. Excel FileTitle of this dataset: Table S2, EpiTYPER resultsDescription of this dataset: Table S2 lists MassARRAY EpiTYPER results. EpiTYPER methylation ratios of individual CpG units in 12 amplicons covering six distinct genomic locations are given for all time course samples of different donors. Click here for file

Additional file 4 **Table S4**. Word DocumentTitle of this dataset: Table S2, Genomic position of analyzed CpG residuesDescription of this dataset: Table S2 lists genomic positions of CpG residues that were analyzed by MassARRAY or QUEST-qPCR. Click here for file

Additional file 5 **Supplementary Figures**. PDFTitle of this dataset: Supplementary FiguresDescription of this dataset: Contains supplementary figures S1-5. Click here for file
