# Supplementary material for: 5-Hydroxymethylcytosine is an essential intermediate of active DNA demethylation processes in primary human monocytes
Source: Genome Biol. 2013 May 26;14(5):R46. doi: 10.1186/gb-2013-14-5-r46 (PMC4053946; doi:10.1186/gb-2013-14-5-r46)
Supplement: Additional file 1 — Table S1. Word DocumentTitle of this dataset: Table S1, Oligonucleotide sequences used for PCRDescription of this dataset: Table S1 lists sequences of oligonucleotides for various PCR-based analyses. [file gb-2013-14-5-r46-S1.DOCX]

**Table S1**

Oligonucleotide primers used for PCR-based assays

| **Gene** | **Primer sequence 5’-3’** (S, sense; AS, antisense) | **Assay** | |
| --- | --- | --- | --- |
| *HOXB1* | S: aggaagagagTTTGAGTTTTATTTGTTTTGGGTGG | MassARRAY ^a^ |  |
|  | AS: cagtaatacgactcactatagggagaaggcTCCTAAAAATACCCTTCCCCAACTC |  |  |
| *HOXB1* | S: CCTCGGAGAGGAGATCAGCA | hMeDIP-qPCR |  |
|  | AS: CCATATCCTCCGCAGCATCC |  |  |
| *MMP7* | S: GCTGTGACATACCTGAGCCTG | hMeDIP-qPCR |  |
|  | AS: CTATGCGACTCACCGTGCTG |  |  |
| *CCL13* | S: ACTTGGTCAACGCCCTGCT | QUEST-qPCR ^b^ |  |
|  | AS: TCTGCTCCTCTGGCTGTTCC |  |  |
| *DNASE1L3* | S: CGCCATCCTCCACAAAGTCAC | QUEST-qPCR |  |
|  | AS: GACCACTTCCGAGTTCATCCAC |  |  |
| *TET1* | S: GCTCTCATGGGTGTCCAATTGCT | qRT-PCR |  |
|  | AS: ATGAGCACCACCATCACAGCAG |  |  |
| *TET2* | S: AAGGCTGAGGGACGAGAACGA | qRT-PCR |  |
|  | AS: TGAGCCCATCTCCTGCTTCCA |  |  |
| *TET3* | S: CCTGCCGATGACAAGCTGGA | qRT-PCR |  |
|  | AS: GAGTTCCCGGATAGAGGCGA |  |  |
| *HPRT1* | S: AAGTTTGTTGTAGGATATGCCC | qRT-PCR |  |
|  | AS: GAACATTGATAATTTTACTGGCG |  |  |

^a^ Mass spectrometry analysis of bisulfite-converted DNA

^b^ Glycosylation assay for the detection of 5hmC
