# Supplementary material for: 5-Hydroxymethylcytosine is an essential intermediate of active DNA demethylation processes in primary human monocytes
Source: Genome Biol. 2013 May 26;14(5):R46. doi: 10.1186/gb-2013-14-5-r46 (PMC4053946; doi:10.1186/gb-2013-14-5-r46)
Supplement: Additional file 3 — Table S3. Excel FileTitle of this dataset: Table S2, EpiTYPER resultsDescription of this dataset: Table S2 lists MassARRAY EpiTYPER results. EpiTYPER methylation ratios of individual CpG units in 12 amplicons covering six distinct genomic locations are given for all time course samples of different donors. [file gb-2013-14-5-r46-S3.DOCX]

**Table S2**

Genomic position of analysed CpG residues

| **Gene** | **Chromosomal Location  (NCBI build 37/hg19)** | **Amplicon (for bisulfite-treated DNA only)** | **Assay** |
| --- | --- | --- | --- |
| *CCL13* | chr17:32683418-32683419 | Epi00109_CCL13.1 ^a^ | MassARRAY ^b^ |
| *STAT5* | chr17:40435680-40435,681 | Epi00104_STAT5A.2 ^a^ | MassARRAY |
| *DNASE1L3* | chr3:58196626-58196627 | Epi00123_DNASE1L3.1 ^a^ | MassARRAY |
| *C9ORF78* | chr9:132601629-132601630 | Epi00147_C9ORF78.2 ^a^ | MassARRAY |
| *MMP7* | chr11:102401438-102401438 | Epi00162_MMP7.1 ^a^ | MassARRAY |
| *HOXB1* | chr17:46607850- 46607851 | Epi00193_HOXB1_01 | MassARRAY |
|  |  |  |  |
| *CCL13* | chr17:32683478-32683479 |  | QUEST-qPCR ^c^ |
| *STAT5* | chr17:40435463-40435464 |  | QUEST-qPCR |
| *DNASE1L3* | chr3:58196930-58196931 |  | QUEST-qPCR |
| *HOXB1* | chr17:46607805-46607806 |  | QUEST-qPCR |

^a^ Amplicons described in Ref. [23]

^b^ Mass spectrometry analysis of bisulfite-converted DNA

^c^ Glycosylation assay for the detection of 5hmC
