# Supplementary material for: 5-Hydroxymethylcytosine is an essential intermediate of active DNA demethylation processes in primary human monocytes
Source: Genome Biol. 2013 May 26;14(5):R46. doi: 10.1186/gb-2013-14-5-r46 (PMC4053946; doi:10.1186/gb-2013-14-5-r46)
Supplement: Additional file 5 — Supplementary Figures. PDFTitle of this dataset: Supplementary FiguresDescription of this dataset: Contains supplementary figures S1-5. [file gb-2013-14-5-r46-S5.PDF]

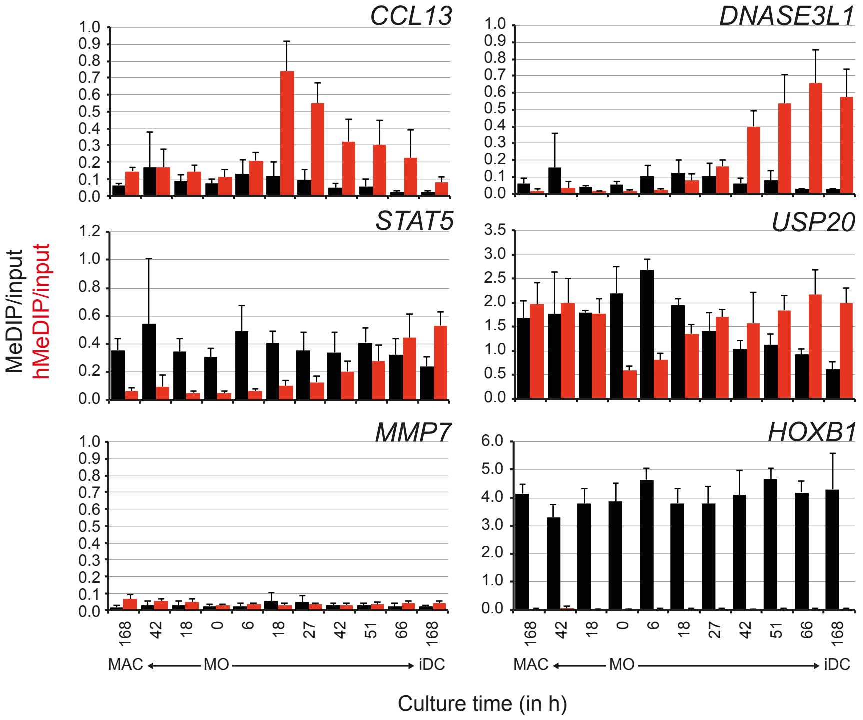

Figure S1

Comparison of hMeDIP and MeDIP results. The same samples were analysed for 5mC and 5hmC enrichment by MeDIP and hMeDIP, respectively. Enrichment is shown relative to the signal observed with the genomic input material. Sensitivities of both antibodies were strikingly different with the 5hmC antibody being much more efficient in precipitating DNA. Especially at low CpG content regions, signals were difficult to quantify. (n=5, values are means ± SD).

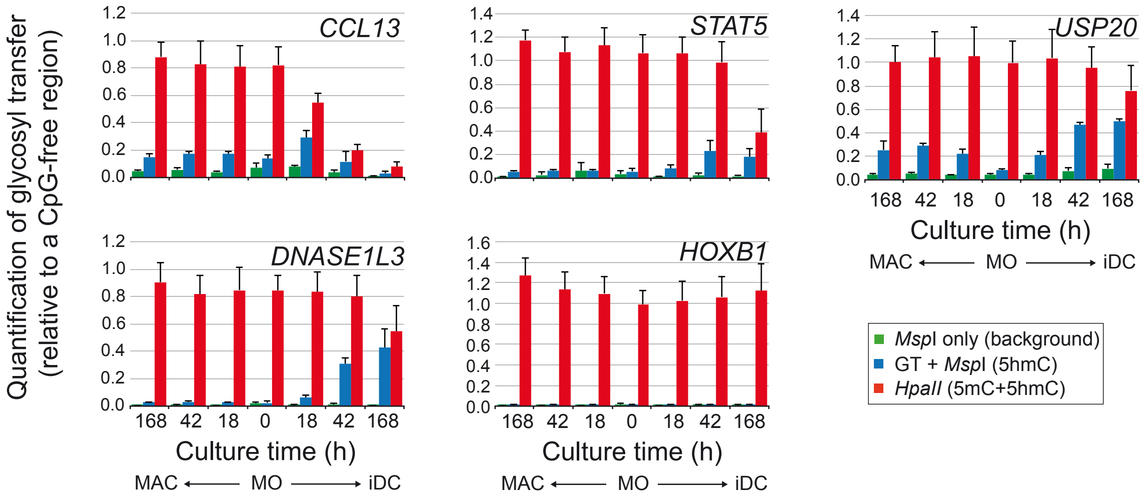

Figure S2

Alternative 5hmC measurements. Quantification of 5hmC using glycosyl transfer to 5hmC followed by glycosylation-sensitve restriction with *MspI* and subsequent amplification of protected regions. DNAs were also digested with *Hpa*II, which is sensitive to both 5hmC and 5mC. QPCR results are shown relative to the amplification of a CpG-free region. Values represent mean ± SD (n≥3) for control digests (*MspI* only), digests after glycosyl transfer (GT+ *MspI*) or digests with *Hpa*II (average for DNAs digested before and after glycosyl transfer). Exact genomic positions of analyzed CpG residues are given in Table S2 in Additional File 2. The *MMP7* region lacks MspI recognition sequences and was not analysed.

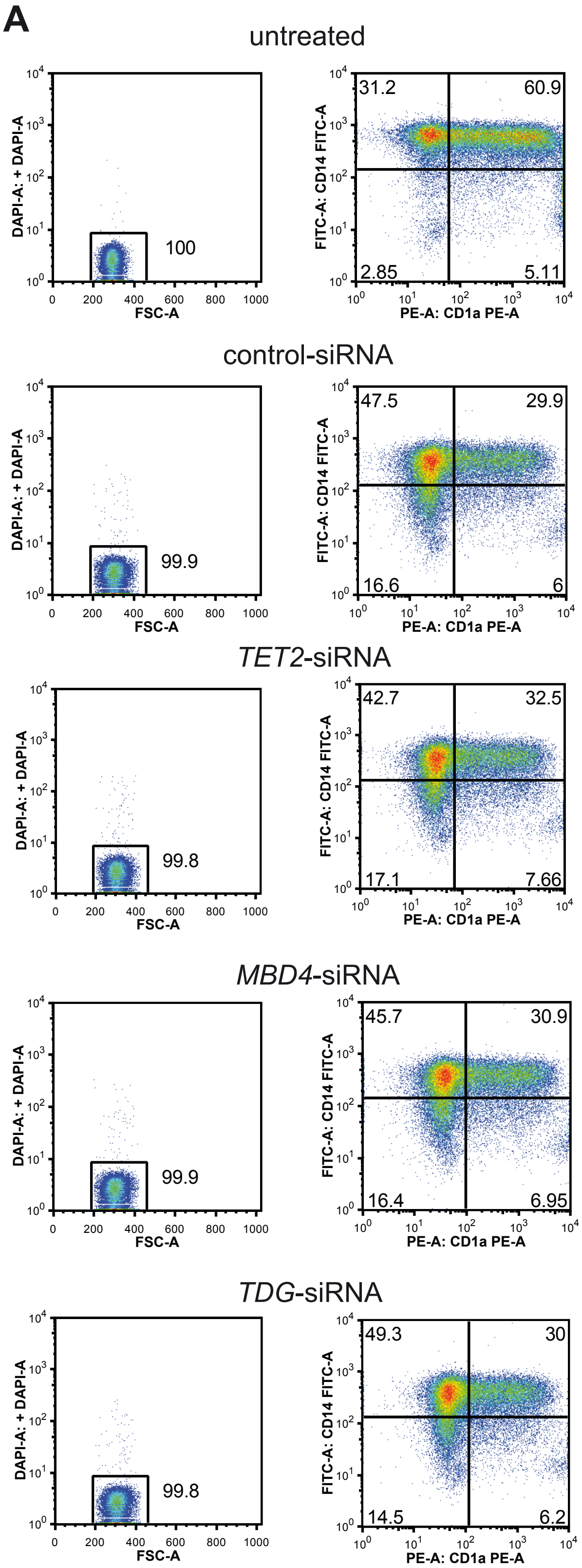

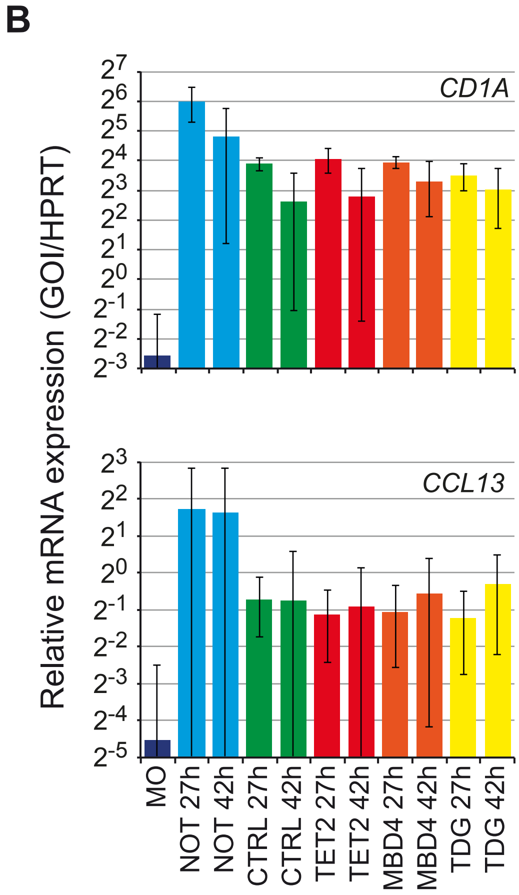

Figure S3

Survival and marker gene expression of siRNA transfected monocytes. (A) Untreated or siRNA-transfected cells after 42h were stained with DAPI (Sigma), anti- CD14-Fitc (My4a, Coulter Clone, a marker for monocytes) and anti-CD1a-PE (T6-RD1; Coulter Clone; a marker for iDC) and analysed by flow cytometry using the LSRII from BD (Heidelberg, Germany). The large majority of cells (>98%) was viable (as demonstrated by the exclusion of DAPI in most cells, top three panels) and entered the DC differentiation pathway (as indicated by the induction of CD1a, right panels), although numbers of differentiating cells were consistently reduced in all transfected samples. (B) Expression of *CD1A* and *CCL13* marker genes in monocytes either non-transfected (NOT) or transfected with TET2-, MBD4-, TDG-siRNA or control siRNA were measured after 27h and 42h of differentiation culture. qRT-PCR results were normalized to *HPRT1* expression. Values represent mean + SD (n≥4).

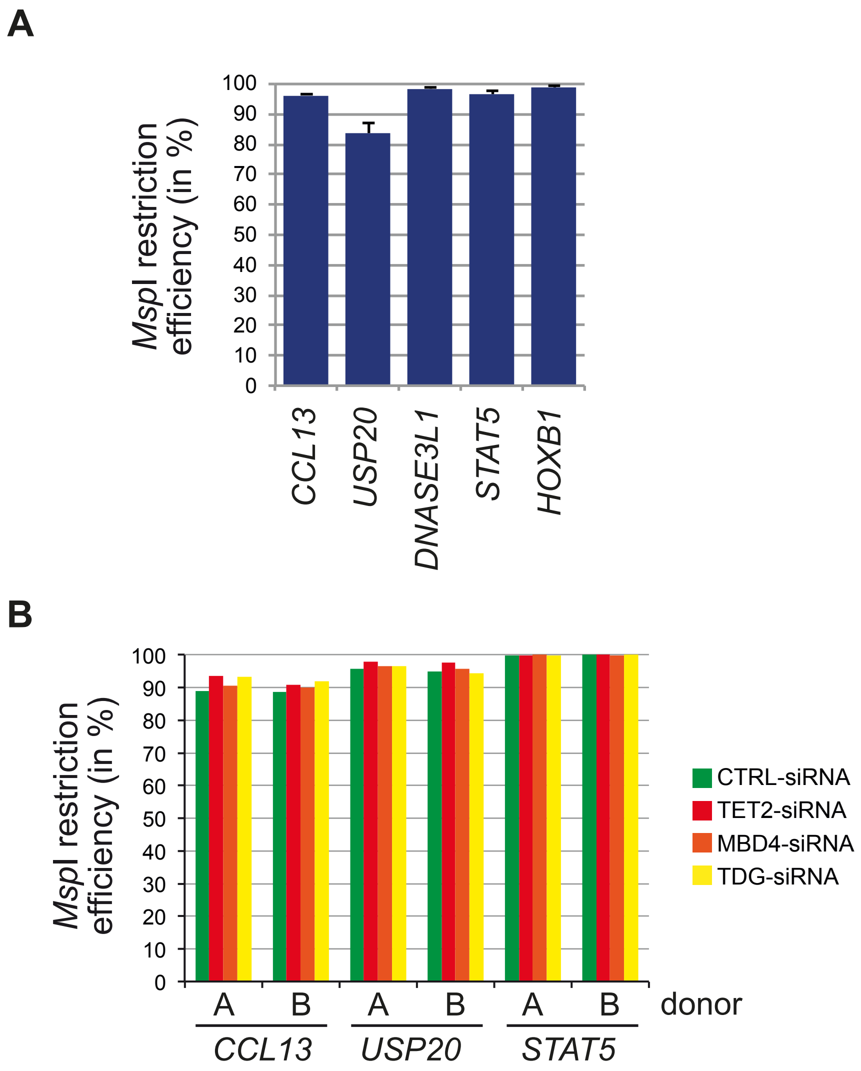

Figure S4

MspI restriction efficiency at demethylated CpG residues. DNA from the indicated untreated (iDC at 7 days of culture, in A) or siRNA transfected cells (27h time point, in B) was subjected to restriction digests as outlined in Materials and Methods. QPCR was applied to all samples to quantify the amplification of fragments amplicons across single MspI sites. Amplification values for the indicated target regions for MspI-digested and control-digested samples were normalized against values obtained for a region in the GAPDH locus lacking an MspI restriction site. Cutting efficiency is calculated by subtracting the ratio (in percent) of normalized values for MspI-digested and control-digested samples from 100%.

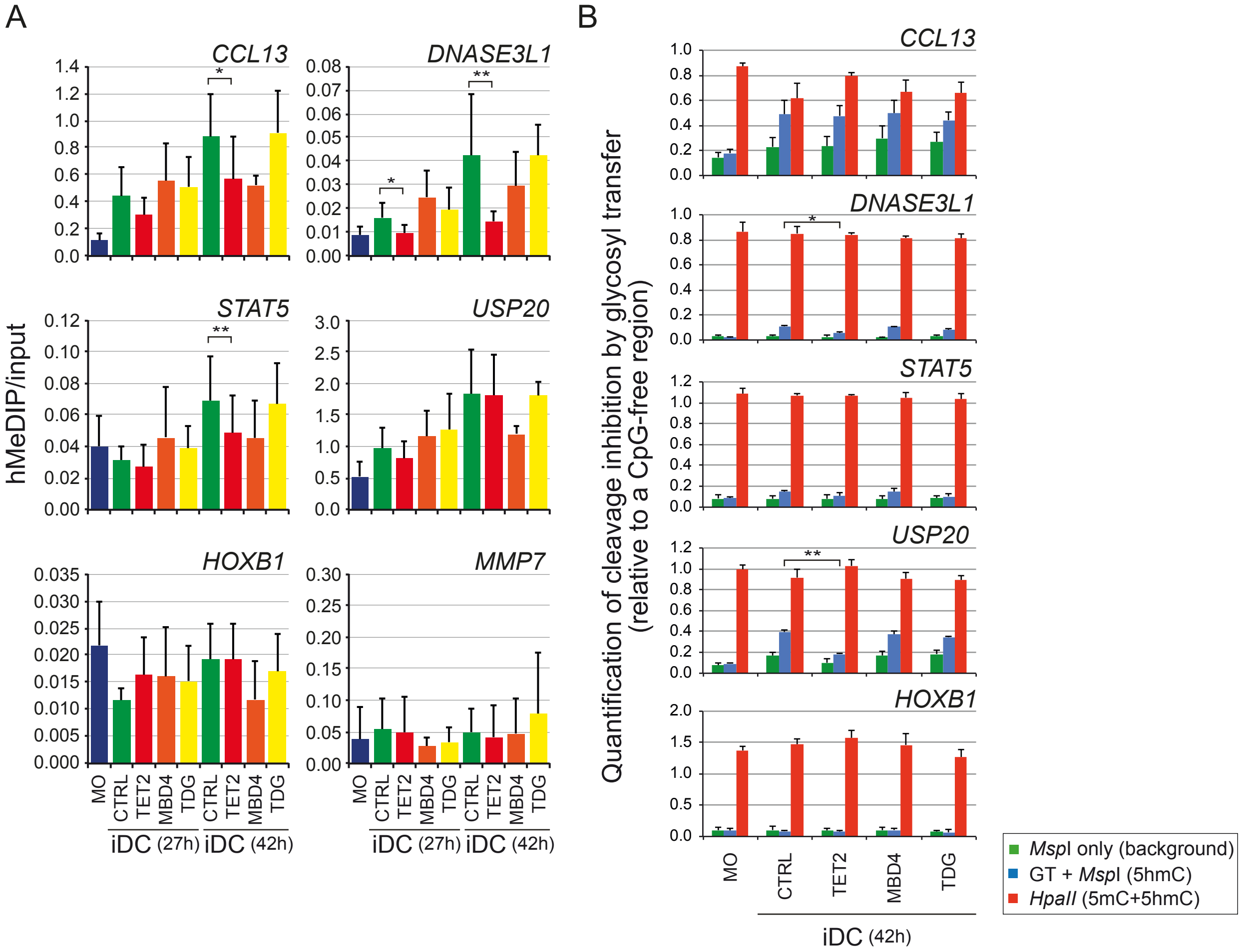

Figure S5

5hmC levels after siRNA-mediated knock-down of TET2, MBD4, and TDG. (A) DNA from the indicated siRNA transfections was subjected to hMeDIP and the enrichment of demethylation targets and control regions was measured using qPCR (n=4, values are means ± SD; * $P<0.05$, ** $P<0.01$ Student's T-test, paired, two-sided). MBD4 values were not significantly different from controls. (B) Quantification of 5hmC using glycosyl transfer to 5hmC followed by glycosylation-sensitve restriction with *MspI* and subsequent amplification of protected regions. DNAs were also digested with *Hpa*II, which is sensitive to both 5hmC and 5mC. QPCR results are shown relative to the amplification of a CpG-free region. Values represent mean ± SD (n=3) for control digests (*MspI* only), digests after glycosyl transfer (GT+ *MspI*) or digests with *Hpa*II (average for DNAs digested before and after glycosyl transfer), which reflects the presence of both 5mC and 5hmC. Exact genomic positions of analyzed CpG residues are given in Table S2 in Additional File 2. The *MMP7* region lacks *Msp*I/*Hpa*II recognition sequences and was not analysed.
